# A New Insight on the Role of the Cerebellum for Executive Functions and Emotion Processing in Adults

**DOI:** 10.3389/fneur.2020.593490

**Published:** 2020-12-23

**Authors:** Pierre-Aurélien Beuriat, Shira Cohen-Zimerman, Gretchen N. L. Smith, Frank Krueger, Barry Gordon, Jordan Grafman

**Affiliations:** ^1^Cognitive Neuroscience Laboratory, Brain Injury Research, Shirley Ryan Ability Lab, Chicago, IL, United States; ^2^Feinberg School of Medicine, Northwestern University, Chicago, IL, United States; ^3^Department of Pediatric Neurosurgery, Hôpital Femme Mère Enfant, Hospices Civils de Lyon, Lyon, France; ^4^Rockfeller School of Medicine, Claude Bernard University, Lyon, France; ^5^School of Systems Biology, George Mason University, Fairfax, VA, United States; ^6^Department of Psychology, University of Mannheim, Mannheim, Germany; ^7^Department of Neurology, Johns Hopkins University School of Medicine, Baltimore, MD, United States; ^8^Department of Cognitive Science, Johns Hopkins University, Baltimore, MD, United States; ^9^Departments of Neurology, Psychiatry, and Cognitive Neurology and Alzheimer's Disease, Feinberg School of Medicine, Northwestern University, Chicago, IL, United States

**Keywords:** cerebellum, executive functions, emotion, brain network, traumatic brain injury

## Abstract

**Objective:** We investigated whether the cerebellum plays a critical or supportive role in in executive and emotion processes in adults. Many investigators now espouse the hypothesis that participants with cerebellar lesions experience executive functions and emotions (EE) disorders. But we hypothesized that these disorders would be milder if the damage is relatively limited to the cerebellum compared to damage involving the cerebellum plus additional cortical areas.

**Methods:** We studied veterans with penetrating Traumatic Brain Injury (pTBI) participating in the Vietnam Head Injury Study (VHIS). We selected veterans with a cerebellar lesion (*n* = 24), a prefrontal cortex lesion (*n* = 20), along with healthy controls (HC) (*n* = 55). Tests of executive functions and emotions were analyzed as well as caregiver burden. We performed between-group null hypothesis significance testing, Bayesian hypothesis tests and correlational analyses.

**Results:** Performance of participants with cerebellar lesions which extended to the cerebral cortex was similar to the HC on the Executive Function tests but they were significantly impaired on the Working Memory Index. No differences were found on the emotional processing tasks with one exception—the Facial Expression of Emotion-Test (FEEST). We then examined a sub-group of participants with large cerebellar lesions (>15%) but minimal lesions in the cerebral cortex (<15%). This sub-group of participants performed similarly to the HC on the Working Memory Index and on the FEEST.

**Conclusions:** We suggest that the cerebellar cortex may not be critical for executive functions or processing emotional stimuli in adults as suggested. Instead, we find that the cerebellum has a supportive role characterized by its computing of the motor requirements when EE processing is required.

## Introduction

Does the cerebellum play a crucial or supportive role in the functions of the cognitive and emotional networks? While the cerebellum's membership in brain networks that extend to various regions of the cerebral cortex has been mapped out, its role in executive function and emotion (EE) processes is unclear.

Traditionally, the cerebellum has been associated with motor control (1). However, recent research suggests that the cerebellum is also crucial in processing higher-order functions such as EE (1). Its connectivity with the Prefrontal Cortex (PFC), which has a major role in these functions (2), *via* cortico-cerebellar loops support this role (3). However, other studies suggest a minimal role of the cerebellum in executive functioning (4–7).

Here, we studied subjects from the Vietnam Head Injury Study (VHIS), a prospective, long-term follow-up study of male Vietnam war veterans with non-lethal penetrating traumatic brain injuries (TBI) mostly due to low velocity shell fragments (typically missile fragments or gunshots; direct bullet wounds were rare in this sample) (8). In addition, we compared our participants to a healthy control group (HC) of combat veterans without a history of neurological disorders.

We anticipated that participants with cerebellar damage would demonstrate executive and/or emotional function disorders. But we expected that these disorders would be milder in participants with a pTBI relatively limited to the cerebellum compared to those with a pTBI involving the cerebellum plus additional cortical areas.

## Materials and Methods

### Participants

Participants were veterans, who participated in the VHIS ~35 years post-pTBI, during Phase 3 (2003–2008) (8). The different groups were compared on key demographic variables including age, sex, education, and war experiences. Participants were young US army soldiers who were healthy and approved for participation in combat at the time of their injury. Unlike patients with other kinds of neurological disorders (e.g., stroke), they did not suffer from neurological associated comorbidity at the time of the pTBI. Moreover, the different groups did not differ in their post-war medical history (hypertension, diabetes, alcohol, subsequent trauma, among others).

All participants understood the study and gave written informed consent, as approved by the National Institutes of Health Neuroscience Institutional Review Board, Bethesda Naval Hospital and Department of Defense Institutional Review Boards. The Institutional Review Board at Northwestern University approved the protocol encompassing the current analysis.

### Methods

#### Neuropsychological Testing

Participants were administered a variety of neuropsychological tests (8). Because executive functioning is multifaceted, no single executive function test is adequate to assess all these processes. Thus, five subtests of the Delis-Kaplan Executive Function System (D-KEFS) were analyzed (the Trail Making-Test, the Verbal Fluency-Test, the Twenty Question-Test, and the Tower-Test) as well as the Wechsler Adult Intelligence Scale IV (WAIS-IV) working memory index score.

Emotion was measured using the Facial Expression of Emotion-Test (FEEST), Mayer-Salovey-Caruso Emotional Intelligence-Test (MSCEIT) and the Vocal Emotional Task.

Descriptions of the different neurobehavioral tests are detailed in the Supplementary Methods.

#### Additional Neuropsychological Testing

Other neuropsychological tests analyzed included the Armed Forces Qualification-Test (AFQT-7A, 1960), a standardized test which is highly correlated with Wechsler intelligence test IQ scores and hence can be used as a surrogate for IQ (9). Pre-injury AFQT scores were obtained upon enlistment prior to service in Vietnam and the same AFQT was re-administered during the Phase 3 evaluation. This additional testing allowed us to ensure that all groups were comparable on a general measure of intellectual functioning.

As part of our study was focused on the emotional consequences of cerebellar damage, we wanted to ensure that the participants from the different groups did not differ in terms of any post-war psychological consequences that could have influenced their emotional processing. Therefore, we also examined any group differences in the diagnosis of Post-Traumatic Stress Disorder (PTSD) as evaluated by a Clinician Administered PTSD Scale for DSM-IV (CAPS-DX).

#### Assessment of Caregiver Burden and Complaints

Previous VHIS studies have shown that if a function is significantly impaired, it affects caregiver burden and complaints (10, 11). Therefore, studying caregiver burden and complaints can illustrate whether any observed deficits in cerebellar patients were notable enough to burden caregivers. The Zarit Burden Interview and the Frontal Systems Behavior Scale (FrSBE) were used to evaluate cognitive and behavioral problems based on a significant other's observations. The different tests are detailed in the Supplementary Methods.

Caregivers were close family members (e.g., spouse/offspring/parents) who volunteered to complete the Zarit Burden scale.

#### Grouping

**Figure 1:**
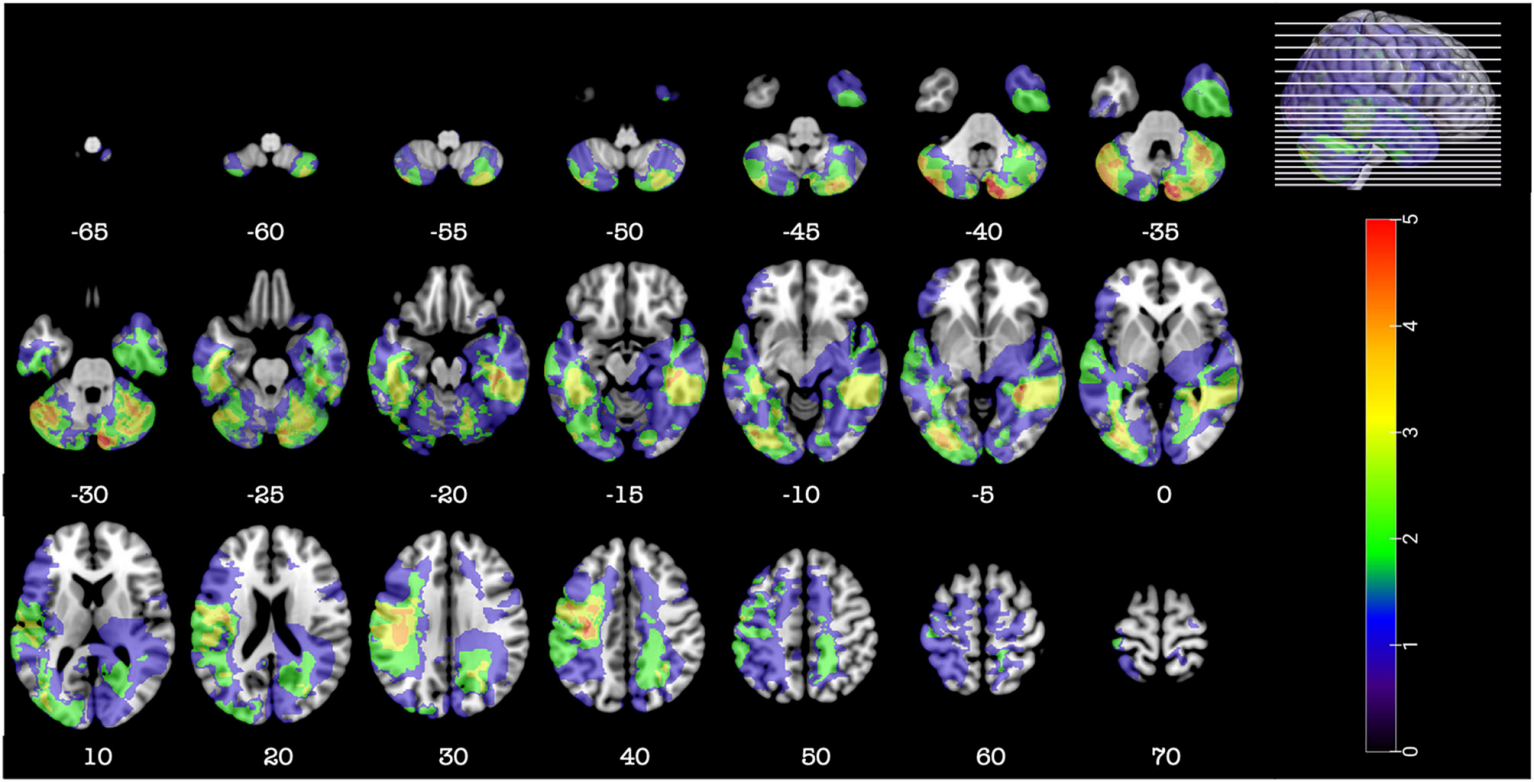
Representative axial slices depicting the lesion overlay density maps of TBI participants in the cerebellar group (*n* = 24). *Z*-values shown at the bottom of each slice indicate the z coordinates (MNI) of each axial slice represented in the 3D view of the brain by white line. The color indicates the number of veterans in the group with damage to a given voxel. Images are in radiological space (i.e., right is left).

For our first set of analyses, from the entire Phase 3 pTBI sample (*n* = 194), we selected a group with a cerebellar lesion (*n* = 24), along with the entire group of HC (*n* = 55). Note that this overall cerebellar group included subjects with pTBIs not restricted to the cerebellum. Lesions may have included the frontal, temporal, parietal, and occipital cortex (Figure 1). No participants were excluded from this group regardless of the percentage of the cerebellar volume loss or the anatomical localization of the lesion (anterior or posterior cerebellum). The exact anatomical localization of the damage to the cerebellar cortex for each participant is detailed in Table 1. Most of the lesions were localized in the posterior cerebellum and no participant only had an anterior cerebellar lesion. Therefore, we could not dissociate an anterior vs. posterior lesion in the statistical analysis. Since damage to the dentate nuclei (DN) is a key factor limiting motor and cognitive recovery, we determined its involvement for each participant using the participant lesion mask applied to the high definition T2 MNI template of MRIcroGL v12 (12) that we used for lesion location identification. The DN was partially involved in only two participants (Supplementary Figure 1).

**Table 1:** Detail of the anatomical localization of the cerebellar injury.

**Participant**	**Cerebellar anatomical label**		
	**Anterior cerebellum**	**Posterior cerebellum**	**Vermis**
1^*^	R IV-V	R VI, R Crus I, R Crus II, R VIIb, R VIII	IV-V, VI
2^*^		L VI, R VI, L Crus I, R Crus I, L Crus II, R Crus II, L VIIb, R VIIb, L VIII, R VIII, R IX	VI, VII, VIII
3		L VI, L Crus I	VI, VII
4^*^	L IV-V	L VI, L Crus I, L Crus II, L VIIb, L VIII, L IX, L X	
5	L IV-V, R IV-V	L VI, R VI, L Crus I, L Crus II, L VIIb, L VIII, L IX	IV-V, VI, VII, VIII
6		R VI, R Crus I	
7		L VI, L Crus I	
8		L Crus I	
9	L III, L IV-V	L VI, L Crus I, R Crus I, R Crus II, L VIIb, L VIII, L IX	IV-V, VI, VII, VIII
10^*^	L IV-V	L VI, R VI, L Crus I, R Crus I, L Crus II, R Crus II, L VIIb, L VIII	VII
11^*^		R VI, R Crus I, R Crus II, R VIIb, R VIII	
12	L III, L IV-V	L VI, L Crus I, L Crus II	III, IV-V
13^*^		R Crus I, R Crus II, R VIIb, R VIII	
14		R VI	VI
15^*^	L IV-V	L VI, L Crus I, L Crus II, L VIIb, L VIII, L IX, L X	
16		L Crus I	
17^*^		R Crus I, R Crus II, R VIIb, R VIII	
18		L VIIb, L VIII	VIII
19^*^		L VI, R VI, L Crus I, R Crus I, L Crus II, R Crus II, L VIIb, R VIIb, L VIII, R VIII, L IX, R IX	VI, VII, VIII, IX
20		R VI, R Crus I	
21	R III, R IV-V	R VI, R Crus I	
22	L IV-V	R VI	IV-V
23	R IV-V	R VI, R Crus I, R Crus II	
24		R Crus I	

*R, Right; L, Left*.

* *Participant included in the cerebellar sub-group with limited lesion to the cortex*.

Comparison of motor behavior utilized a clinical motor evaluation (Paresis, Ataxia, Gait Abnormalities, Voluntary Movement Abnormalities, Station Posture Impairment, Muscle Tone Impairment) and the Purdue Pegboard score (Both Hands and Assembly score).

**Figure 2:**
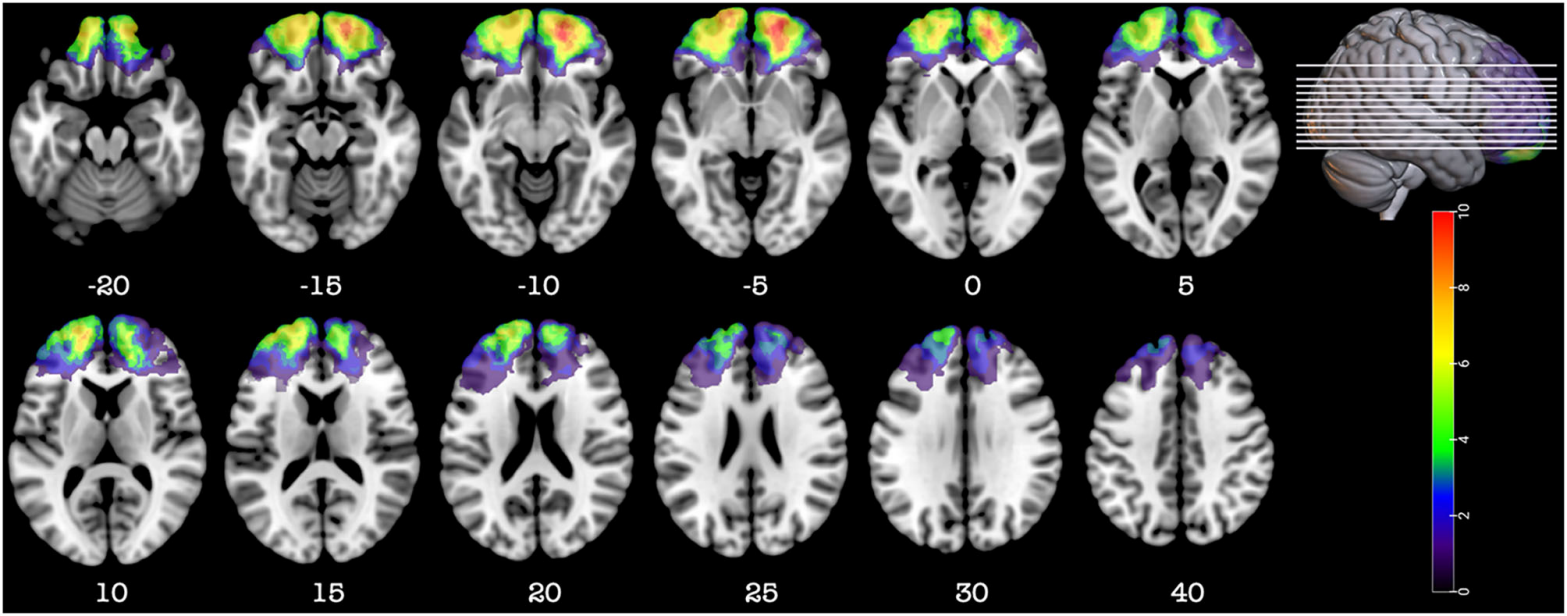
Representative axial slices depicting the lesion overlay density maps of TBI participants in the prefrontal group (*n* = 20). *Z*-values shown at the bottom of each slice indicate the z coordinates (MNI) of each axial slice represented in the 3D view of the brain by white line. The color indicates the number of veterans in the group with damage to a given voxel. Images are in radiological space (i.e., right is left).

We then compared the cerebellar lesion and HC groups to a group of subjects with PFC pTBIs (*n* = 20). PFC lesions included the ventromedial and dorsomedial and lateral prefrontal cortex (Figure 2). We used this comparison group because frontal lobe lesions are known to impair EE functions allowing us to place in perspective any observed deficits in the cerebellar participants.

**Figure 3:**
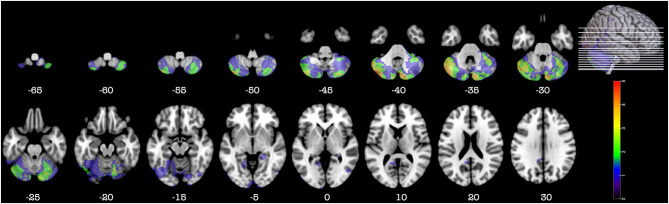
Representative axial slices depicting the lesion overlay density maps of TBI participants in the sub-group of cerebellar participants with a large cerebellar lesion (>15%) but a small lesion (<15%) in other part of the brain (*n* = 9). *Z*-values shown at the bottom of each slice indicate the z coordinates (MNI) of each axial slice represented in the 3D view of the brain by white line. The color indicates the number of veterans in the group with damage to a given voxel. Images are in radiological space (i.e., right is left).

To ensure that our results were specific to cerebellar injury, we selected, from the overall cerebellar group, a subgroup of cerebellar participants with a lesion >15% within the cerebellum (cerebellar volume lesion in cc^3^: average 17.1; min: 4.97; max: 29.07) and <15% in other part of the brain (*n* = 9) (Figure 3). Since small lesion volumes are more amenable to recovery of function (13), we set this relatively high cerebellum volume loss threshold, above which it was likely that any acquired impairments would still be present at Phase 3, some 30+ years after the original injury. Moreover, in order to eliminate the potential involvement of cortical damage, we also set the same volume loss threshold to exclude participants with large cortical lesions. Past work has demonstrated that >15% damage to a target brain region can be sufficient to induce lasting deficits (14). The overall coverage of the cerebellum in this subgroup was not different than in the overall cerebellar group. Only measures that indicated the participants of the overall cerebellar group performed worse than controls were reanalyzed within the subgroup.

Finally, we used MRIcroGL V12 to compare the overlay maps of a past study of our group on the role of the parietal cortex in EF (15) with the current study's overall cerebellar group to help decipher the role of cortical lesions in addition to the cerebellar injury. Description of the population of the study on the role of the parietal cortex in EF has been previously reported (15).

### CT Image Acquisition and Analysis

Detail of the axial computed tomography (CT) acquisition is described in the Supplementary Methods. Since metal was retained in the brain due to penetrating wounds or surgical materials, MRI scans could not be acquired. Localization and analyses of the lesion were done as described in the Supplementary Methods. Figures 1–3, 5 were constructed using MRIcroGL v12 (12).

### Statistical Analyses

We performed null hypothesis significance testing. Significance level was set to *p* < 0.05 (one-tailed since we anticipated that cerebellar participants would be expected to perform worse than controls). A Bonferroni correction was applied when multiple comparisons were performed. We checked normality of data using the Shapiro-Wilk-test and homogeneity of variance using Levene's-test and conducted parametric [one-way analysis of variance (ANOVA) and independent *t*-tests] or non-parametric (Kruskal-Wallis and Mann-Whitney U-tests) statistical tests as appropriate. Spearman's rank-order correlations were also performed. We supplemented the standard statistical analyses with Bayesian hypothesis tests (Bayesian independent sample *t*-test, Bayesian ANOVA and Bayesian correlation) as recent advances in quantitative psychology have criticized the practice of completely relying on *p*-values for evidence (16–19). Bayesian analysis has several advantages. It provides a comparison between a null (H0) and alternative hypothesis (H1) and can quantify the evidence in favor of one or the other. Moreover, no bias exists against the null hypothesis.

All analyses were carried out using the JASP software package 0.11.1 (20). Details on the Bayesian analyses interpretation are described in the Supplementary Methods.

## Results

### Demographic and Motor Group Comparison

There was no significant differences between the cerebellar group, the PFC group and control group with respect to age (F_(2, 96)_ = 0.724, *p* = 0.49, η^2^ = 0.015), total years of education [F_(2, 95)_ = 1.312, *p* = 0.27, η^2^ = 0.027], handedness [F_(2, 96)_ = 1.205, *p* = 0.3, η^2^ = 0.024], pre-injury AFQT [F_(2, 73)_ = 1.517, *p* = 0.23, η^2^ = 0.04], post-injury AFQT [F_(2, 71)_ = 1.873, *p* = 0.16, η^2^ = 0.05] and PTSD [F_(2, 95)_ = 1.253, *p* = 0.29, η^2^ = 0.026] (Table 2). The group of cerebellar participants showed significant motor impairment compared to HC (Supplementary Table 1). No rank-order correlations were found between the Purdue Pegboard score and overall cerebellar lesion volume nor the percentage of damage to each cerebellar structure.

**Table 2:** Demographics for combat veterans with cerebellar and PFC damage and combat veterans without a history of neurological disorder (Healthy control).

	**Cerebellar group (*n* = 24)**	**PFC group (*n* = 20)**	**Healthy control (*n* = 55)**	**Statistics**
Age	*M* = 58.2 (SD = 2.61)	*M* = 58 (SD = 4.6)	*M* = 59 (SD = 3.4)	F_(2, 96)_ = 0.724, *p* = 0.49, η^2^ = 0.015
Education	*M* = 14.83 (SD = 2.16)	*M* = 14.82 (SD = 2.27)	*M* = 15.19 (SD = 2.47)	F_(2, 95)_ = 1.312, *p* = 0.27, η^2^ = 0.027
Handedness (R, L, A)	21, 3, 0	16, 3, 1	43, 8, 4	F_(2, 96)_ = 1.205, *p* = 0.3, η^2^ = 0.024
Pre-injury AFQT	*M* = 55.05 (SD = 26.3)	*M* = 56.7 (SD = 23.35)	*M* = 65.40 (SD = 22.91)	F_(2, 73)_ = 1.517, *p* = 0.23, η^2^ = 0.04
(Post-injury)-(Pre-injury) AFQT	*M* = −4.86 (SD = 23.8)	*M* = 3.42 (SD = 12.63)	*M* = 3.92 (SD = 14.47)	F_(2, 71)_ = 1.873, *p* = 0.16, η^2^ = 0.05
CAPS-DX (Y, N, M)	8, 16, 0	7, 13, 0	27, 27, 1	F_(2, 95)_ = 1.253, *p* = 0.29, η^2^ = 0.026

*PFC, Pre-Frontal Cortex; R, Right; L, Left; A, Ambidextrous; RBC, Rank-Biserial Correlation; M, Mean; SD, Standard Deviation; AFQT, Armed Forces Qualification Test; CAPS-DX, Clinician Administered PTSD Scale for DSM-IV; Y, Yes; N, No; M, Missing*.

*Age and education are listed in the number of years*.

### Behavioral Analyses

#### Executive Function

Cerebellar participants performance on the 5 D-KEFS subtests was similar to the HC (Supplementary Table 2) using Bonferroni adjusted alpha levels of 0.0125 per test (0.05/4).

In contrast, cerebellar participants were found to be significantly impaired compared to HC on the WAIS-IV Working Memory Index (Mcereb = 97.67, SDcereb = 16.22, Mcontrol = 105.75, SDcontrol = 12.45, *U* = 426.5, *p* = 0.014, RBC = −0.317). Note that the mean scores of both groups were in the normal range. The corresponding Bayesian two-sample *t*-tests confirmed H1 (BF_10_ = 5.34) (H1: WAIS-IV Working Memory Index Score differs between cerebellar participants and HC).

A rank-order correlation between the WAIS-IV Working Memory Index and the overall cerebellar lesion volume loss was not significant (Spearman's rho = 0.188, *p* = 0.8). Rank-order correlations using the percentage of damage of each cerebellar structure revealed a significant negative correlation between the Weschler Adult Intelligence Scale-IV Working Memory Index and percentage of damage to Vermis III (Spearman's rho = −0.347, *p* = 0.048, BF_10_ = 1.81). But the corresponding Bayesian correlation was not supportive of H1 (BF_10_ = 1.81) (H1: the percentage of damage of the designated anatomical cerebellar structure is correlated with the WAIS-IV Working Memory Index score).

Finally, rank-order correlations revealed a significant association between the WAIS-IV Working Memory Index score and motor performance using the Purdue Pegboard both hands and Assembly measures (Spearman's rho = 0.1484, *p* = 0.015 and Spearman's rho = 0.422, *p* = 0.032 respectively). The corresponding Bayesian correlation confirmed H1 for the Pegboard both hands score only (BF_10_ = 3.223) (H1: WAIS-IV Working Memory Index Score is correlated with motor performance using the Purdue Pegboard both hands score). However, it did not support H1 (BF_10_ = 1.861) for the Purdue Pegboard Assembly score (H1: WAIS-IV Working Memory Index Score is correlated with motor performance using the Pegboard Assembly score).

#### Emotions

On the FEEST, cerebellar participants were significantly impaired compared to HC (Mcereb = 96.26, SDcereb = 16.04, Mcontrol = 104.62, SDcontrol = 10.38, *U* = 416.0, *p* = 0.014, RBC = −0.317). The corresponding Bayesian two-sample *t*-tests confirmed H1 (BF_10_ = 5.38) (H1: FEEST Score differs between cerebellar participants and HC).

Results from the Mayer-Salovey-Caruso-Emotional-Intelligence-Test are summarized in Supplementary Table 3. No significant differences were found between cerebellar participants and HC using Bonferroni adjusted alpha levels of 0.007 per test (0.05/7).

On the Vocal Emotion Task, the cerebellar group performed similarly to the HC (Mcereb = 0.351, SDcereb = 0.09, Mcontrol = 0.339, SDcontrol = 0.08, *U* = 422.5, *p* = 0.589, RBC = 0.036, BF_10_ = 0.237).

### Cognitive and Behavior Burden and Complaints by Relatives

Results from the Zarit Burden Interview and the FrSBE are summarized in Supplementary Table 4. No significant differences were found between the caregivers of cerebellar participants and HC on either of the measures using Bonferroni adjusted alpha levels of 0.0125 per test (0.05/4 for the FrSBE).

### Additional Analyses

We next compared the cerebellar group, the HC group and the group of participants with pure PFC pTBI.

**Figure 4:**
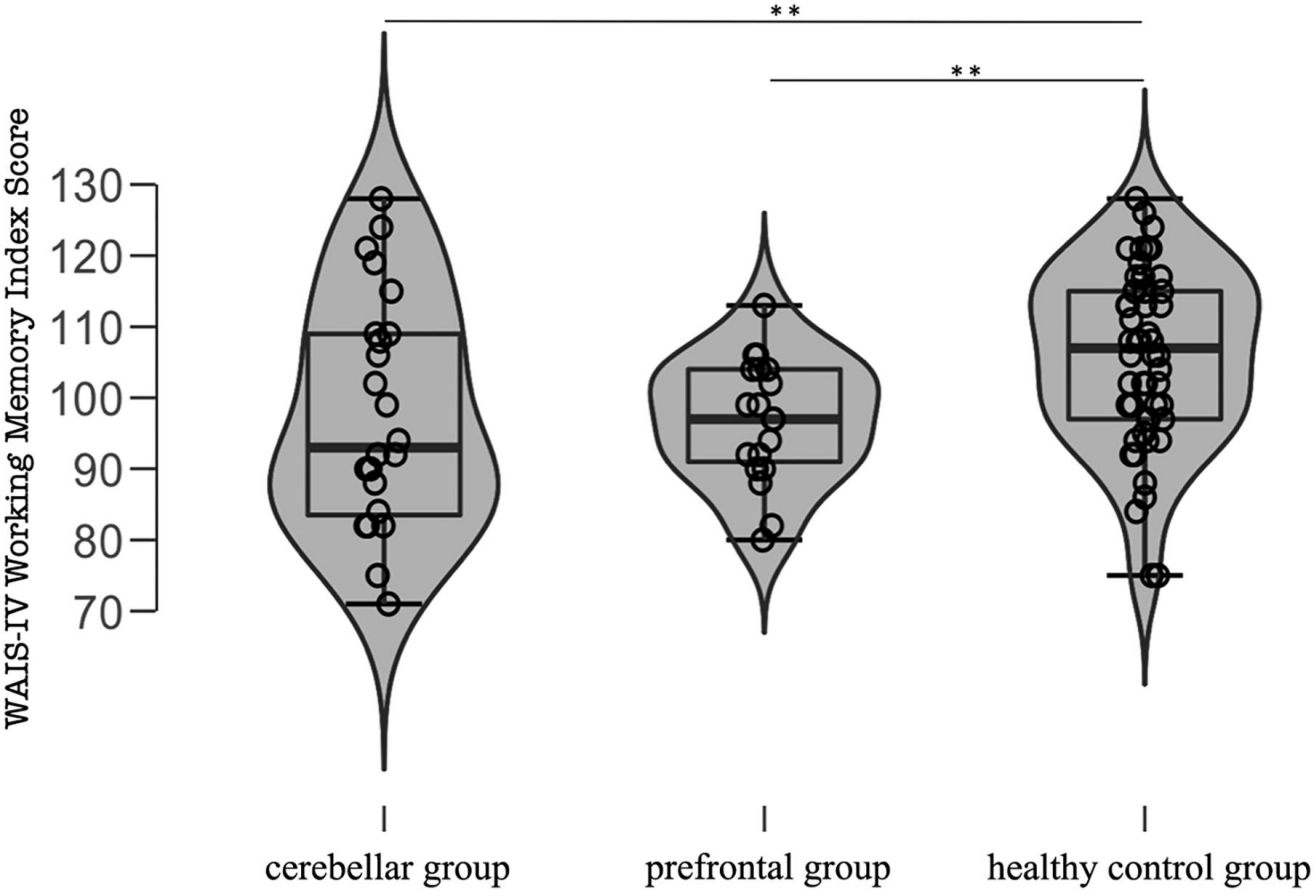
Violin plots, sample size and individual data points of the three groups (cerebellar group; prefrontal group; HC group) of the Weschler Adult Intelligence Scale IV (WAIS-IV) working memory index score. **Significantly different *p* < 0.05.

For analysis of the WAIS-IV Working Memory Index score, the Kruskal-Wallis-test was used, revealing a significant overall difference [$\chi_{\left(2,n=95\right)}^{2}$ = 10.31, *p* = 0.006] (Figure 4). The corresponding Bayesian ANOVA confirmed H1 (BF_10_ = 6.296) (H1: WAIS-IV Working Memory Index Score differs between groups (cerebellar group, prefrontal group and HC). Follow up Mann-Whitney-tests showed that the cerebellar group scored lower than the HC group (*U* = 426.5, *p* = 0.014, RBC = −0.317). The corresponding Bayesian two-sample *t*-tests confirmed H1 (BF_10_ = 5.34) (H1: WAIS-IV Working Memory Index Score differs between cerebellar participants and HC). The cerebellar group, however, did not perform significantly different than the prefrontal group (*U* = 227.5, *p* = 1.00, RBC = −0.002, BF_10_ = 0.31) on this measure. As expected, the prefrontal group scored lower than the HC group (*U* = 266.5, *p* = 0.002, RBC = −0.461). The corresponding Bayesian two-sample *t*-tests confirmed H1 (BF_10_ = 7.89) (H1: WAIS-IV Working Memory Index Score differs between prefrontal participants and HC).

### Cerebellar Sub-group Analyses

There was no significant differences between the cerebellar subgroup (*n* = 9) and control group (*n* = 55) with respect to age (*U* = 115.0, *p* = 0.237, RBC = 0.278), total years of education [t_(27)_ = 0.511, *p* = 0.61, *d* = 0.205], handedness [${\text{X}}_{\left(2,N=29\right)}^{2}$ = 0.577, *p* = 0.75], pre-injury AFQT [t_(25)_ = 0.041, *p* = 0.97, *d* = 0.018] and post-injury AFQT [t_(24)_ = 0.484, *p* = 0.63, *d* = 0.214].

On the WAIS-IV Working Memory Index score, this sub-group of cerebellar subjects performed worse than HC [Mcereb = 99.33, SDcereb = 12.19, Mcontrol = 105.75, SDcontrol = 12.45, t_(60)_= −1.513, *p* = 0.068, *d* = −0.55] with both groups' means being in the normal range. This was confirmed by the corresponding Bayesian two-sample *t*-tests which did not support H1 (BF_10_ = 1.00) (H1: WAIS-IV Working Memory Index Score differs between the sub-group of cerebellar subjects and HC).

**Figure 5:**
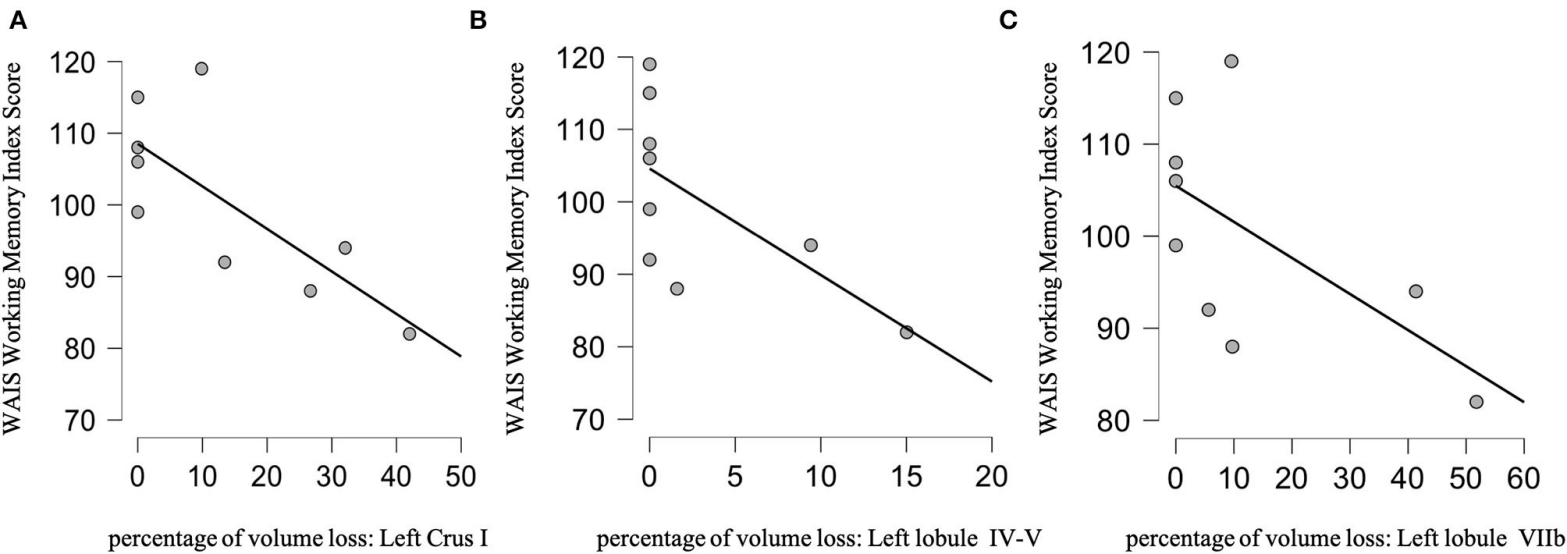
Scatter plots of the significant correlation analysis between the WAIS Working Memory Index Score and the percentage of brain volume loss of cerebellar left Crus I **(A)**, left lobule IV-V **(B)**, and left lobule VIIb **(C)**.

Rank-order correlations examining the relationship between the WAIS-IV Working Memory Index score and the overall cerebellar lesion volume loss were not significant (Spearman's rho = −0.150, *p* = 0.3, BF_10_ = 0.478). Rank-order correlations using the percentage of damage of each cerebellar structure did reveal a negative correlation between the WAIS-IV Working Memory Index score and percentage of brain volume loss to the Left Crus I (Spearman's rho = −0.731, *p* = 0.013, BF_10_ = 9.49), Left Lobule IV-V (Spearman's rho = −0.733, *p* = 0.012, BF_10_ = 3.61) and Left Lobule IV-V (Spearman's rho = −0.627, *p* = 0.035, BF_10_ = 2.95) (Figures 5A–C). The corresponding Bayesian correlation demonstrated substantial support for H1 for damage to the Left Crus I and Lobule IV-V (BF_10_ = 9.49 and 3.61 respectfully) (H1: WAIS-IV Working Memory Index Score is correlated with the percentage of volume loss to these cerebellar structures). However, it did not support H1 (BF_10_ = 2.95) for damage to the Left Lobule VIIb.

For the correlation analysis, we adjusted *p*-values using a Bonferroni Correction of 0.05/8 (0.006) for the vermis (8 differents ROIs), and 0.05/9(0.005) for each cerebellar hemisphere (9 differrents ROIs in each hemisphere). None of the significant correlations survived this Bonferroni correction. However, as we also computed Bayesian statistical analyses on this data and for the left Crus I and lobule IV-V, the BF10 is strong (>3), and support our conclusion.

On the FEEST, the sub-group of cerebellar participants performed similarly compared to HC (Mcereb = 103.44, SDcereb = 14.48, Mcontrol = 104.62, SDcontrol = 10.38, *U* = 229.0, *p* = 0.43, RBC = −0.040). This was confirmed by the corresponding Bayesian correlation which did not support the alternative hypothesis (BF_10_ = 0.478) (H1: FEEST Score differs between the subgroup of cerebellar participants and HC).

The group of the remaining participants with cerebellar lesions but with large cortical ones were also analyzed (*n* = 15).

On the WAIS-IV Working Memory Index score, this sub-group of subjects performed significantly worse than HC (McerebwithsupraTent = 96.07, SDcerebwithsupraTent = 18.31, Mcontrol = 105.75, SDcontrol = 12.45, *U* = 254.5, *p* = 0.02, RBC = −0.347) but with both groups' means being in the normal range. The corresponding Bayesian two-sample *t*-tests did support H1 (BF10 = 5.41) (H1: WAIS-IV Working Memory Index Score differs between the sub-group of cerebellar subjects and HC). Rank-order correlations examining the relationship between the WAIS-IV Working Memory Index score and the overall cerebellar lesion volume loss were not significant (Spearman's rho = 0.151, *p* = 0.6, BF10 = 0.336). On the FEEST, the sub-group of cerebellar participants performed significantly worse than HC [McerebwithsupraTent = 91.643, SDcerebwithsupraTent = 15.74, Mcontrol = 104.62, SDcontrol = 10.38, t_(60)_ = −3.71, *p* < 0.001, *d* = −1.11]. This was confirmed by the corresponding Bayesian correlation which supported the alternative hypothesis (BF10 = 60.204) (H1: FEEST Score differs between the subgroup of cerebellar participants and HC).

### Role of the Parietal Cortex in Working Memory

**Figure 6:**
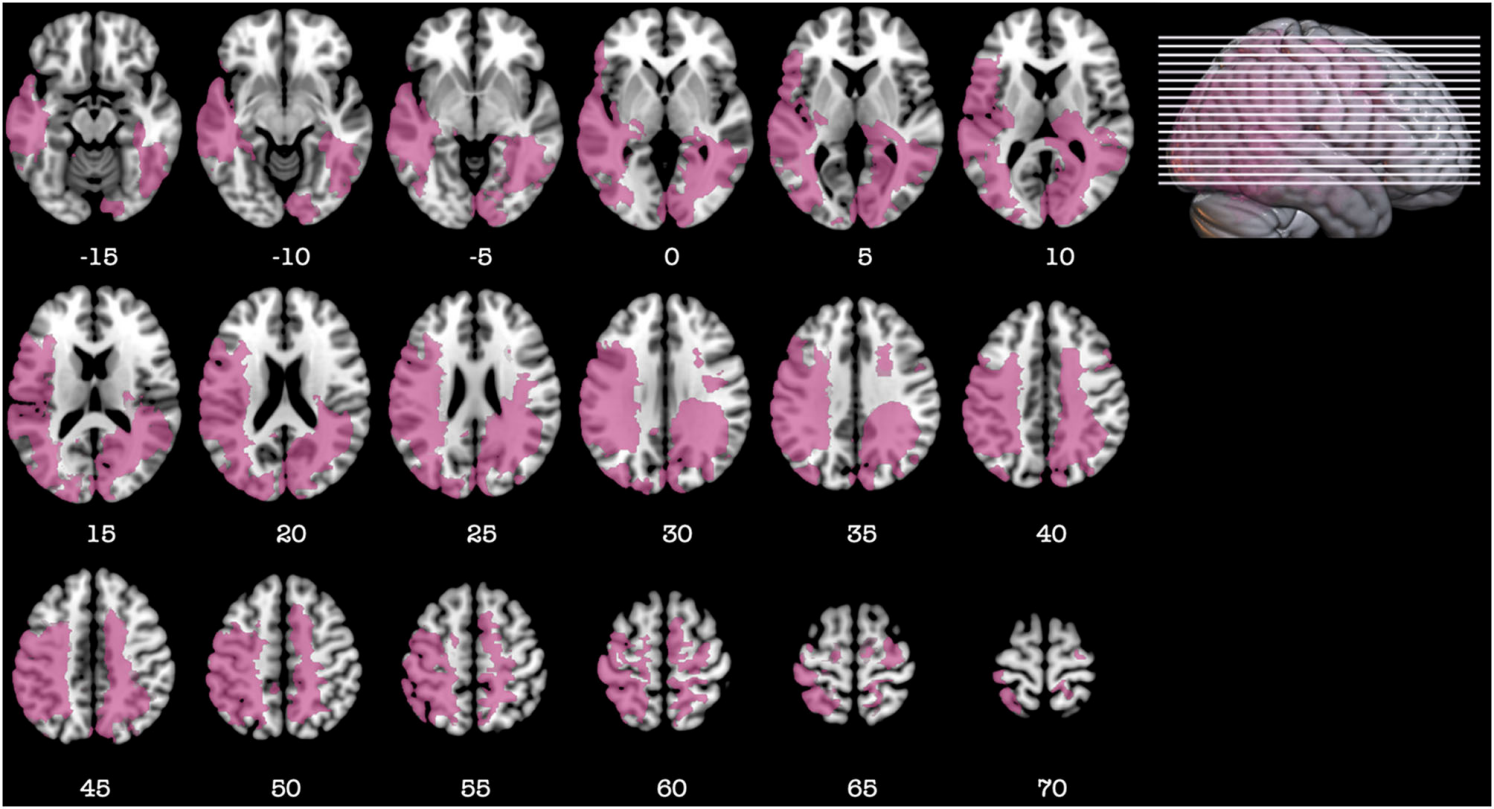
Representative axial slices depicting the anatomical overlap between the participants used in the Koenigs et al. study and in the current study who were members of the cerebellar group with large cortical lesions. Anatomical overlap is represented in pink. *Z*-values shown at the bottom of each slice indicate the z coordinates (MNI) of each axial slice represented in the 3D view of the brain by white line. Images are in radiological space (i.e., right is left).

In 2009, we published a paper showing that injury to the parietal cortex is critical for working memory processing (15). When we overlapped the lesion map of the 2009 patients and the current sample using MRIcroGL, we found a striking anatomical similarity between the Koenigs et al. study and the anatomical localization of the cortical lesions in our current overall cerebellar group (Figure 6). Note that the participants in the two studies were selected from the same VHIS Phase but only two subjects participated in both studies.

### Summary of Results

Cerebellar participants (the group with cerebellar lesions and large cortical ones) performance on the 5 D-KEFS subtests was similar to the HC. Cerebellar participants in the overall cerebellar group were found to be significantly impaired compared to HC on the WAIS-IV Working Memory Index. A rank-order correlation between the WAIS-IV Working Memory Index and the overall cerebellar lesion volume loss was not significant. On the FEEST, cerebellar participants in the overall cerebellar group were significantly impaired compared to HC. On the Vocal Emotion Task, cerebellar participants in the overall cerebellar group performed similarly to the HC. No significant differences were found between the caregivers of cerebellar participants and HC on either of the measures.

On the WAIS-IV Working Memory Index score, the participants in the sub-group of cerebellar participants [group with large cerebellar lesions (>15%) but minimal lesions to the cerebral cortex (<15%)] did not perform worse than HC. In this sub-group of cerebellar participants, Rank-order correlations using the percentage of damage of each cerebellar structure did reveal a negative correlation between the WAIS-IV Working Memory Index score and percentage of brain volume loss to the Left Crus I, Left Lobule IV-V.

## Discussion

This study re-examined the role of the cerebellum in EE processing in adults with a pTBI suffered decades earlier. Those participants, with a cerebellar lesion, performed similarly to HC, so long as they had no, or relatively small supra-tentorial cortical lesions.

Neuropsychological studies of cerebellar patients have reported impairments in every higher order function including functions that are considered EFs [for a review, see (1)] and emotion processes [for a review, see (21)]. Nevertheless, there was variation in the type of patient' etiology and chronic and acute or focal and diffuse pathologies were mixed (22–25). Acute and chronic lesions do not always result in the same deficits even if located in the same anatomical areas (26). There was also considerable variation in the time between the diagnosis of the cerebellar disorders and testing with most of the studies testing subjects within a few weeks or months after the injury [for a review, see (22)] as opposed to the much longer time period in our study. Moreover, when comparing acute phase studies with late phase studies, impairment at the acute phase usually becomes subtle or even disappears after a few weeks [for a review, see (22)] suggesting rapid plasticity or the non-criticality of the cerebellar region for the function investigated. Furthermore, after a posterior fossa stroke or tumor, patients' performance might reflect brain damage outside the cerebellum. They might also experience intracranial hypertension, occasionally hydrocephalus, and brainstem compression (27). Moreover, patients with chronic cerebellar ataxia also experience extra cerebellar neuronal degeneration (28). These phenomena may worsen the test results and deceive the evaluator that the observed impairments were due to the cerebellar damage alone.

Consistent with our findings, there are others reports of mild to no cognitive dysfunction in patients with cerebellar lesions (4, 5, 29). Others reported deficits on certain EF tests in patients compared to controls, but performances were within the normal range (30). In our study, in the overall cerebellar group with the cerebellar lesion extending to the cortex, only working memory and face emotion recognition were impaired but not the other aspects of EF nor emotion processing. Importantly, these impairments were not found in the cerebellar subgroup in which participant had a large lesion of the cerebellar cortex (>15%) but a limited (<15%) lesion to the cerebral cortex. In addition, even on the working memory task in the overall cerebellar group, participants performed within the normal range.

Our results suggest that the cerebellum may not play such a critical role in adult EF and emotional processing as suggested (31). This view is strengthened by the lack of burden or EF complaints reported by the caregivers and by the fact that the group of participants with a cerebellar lesion associated with large cortical ones still experienced some WM and emotional impairments. A supportive, rather than critical, role for the cerebellum in EF and emotions has been suggested before. It was hypothesized that that the cerebellum is the hub in the network that prepares for the neural processing of a stimulus by learning and recognizing an event action sequence through visuospatial detection and then optimizing it. An impairment in this role would lead to a decrease in the overall efficiency (32) of a behavior that utilizes the optimization of event or action sequence processing that is needed on most working memory tasks. In support of this view, and in addition to our study results, a recent study reported that their social/affect task mostly activated a cerebellar region associated with eye movements (33).

We also found that damage to cerebellar lobules IV-V was negatively correlated with working memory scores. Lobules IV-V are part of the anterior cerebellum that, for some authors, is the motor cerebellum, in contrast to the role of the posterior cerebellum in cognitive processing (34). In that conceptualization, it could be argued that the part of the cerebellar lesion that is located in the anterior lobe is disrupting motor control and that the damaged posterior lobe has an effect directly on the cognitive processes underlying working memory. However, the dichotomy of the cerebellum (anterior = motor and posterior = cognitive) is a subject of debate in the literature (35, 36). Indeed, using direct electrical stimulation (DES) to the cerebellar cortex, focal evoked movements were triggered in the anterior and in the posterior cerebellum (35, 36)_._ Moreover, lobules IV-V are not simply connected to PFC but also to the primary motor cortex (3). The primary motor cortex is interconnected with the prefrontal region playing an inhibitory role in motor control (37) processes that might also support working memory maintenance and rehearsal. We did find a correlation between a low working memory index score and cerebellar patients impaired motor control. Is it possible that the cerebellar contribution to working memory is in computing the motor component of ordering and rehearsal? If so, it would point to the cerebellum having a supportive rather than crucial role in working memory. To further add to our hypothesis, we found an anatomical overlap between the parietal lesion of participants of a prior study (15), and our group of cerebellar patients with a large supra-tentorial injury. Because in the Koenigs et al. study, the parietal cortex lesions were associated with working memory impairment (15) and since our results did not indicate a deficit in our cerebellar sub-group (with restricted lesions to the cortex) but impairment in our overall cerebellar group (with a large cortical lesion), it is possible that the additional parietal cortex injury was a critical factor causing our cerebellar participants diminished working memory. Similar hypotheses regarding the role of the parietal cortex for emotion processing, in our cohort, may be suggested. Indeed, neuroimaging studies have attributed a crucial role of the temporo-parietal cortex for the identification and mapping of emotions, in addition to orbital and medial prefrontal cortex (38–40).

Another point to take into consideration is the age of our participants at injury. Some studies reported that cerebellar damage at a young age contributed to more severe, and prolonged impairment in both cognitive and motor domains (41, 42). However, multiple caveats in experimental design limited those studies (43). Beuriat et al., in a study of patients who were treated for a posterior fossa tumor, controlled all the major confounders (namely radiotherapy, tumor characteristics, damage to the deep cerebellar nuclei, and delay between surgery and assessment time) and reported that younger children had worse long-term performance compared to older children or adults in motor and cognitive tasks (43). Since our participants suffered their injury at an adult age, it might also be a key factor that explains our lack of significant findings in our sub-group of cerebellar participants.

Our study has its limitations. We enrolled a small number of participants with pTBI limited to the cerebellum (*n* = 9) which could have precluded obtaining significant results. Secondly, the participants were mostly white Americans and all were male military combat veterans with a pTBI, which would limit generalizing our findings to other populations. The use of CTs is another limit of this study. However, high definition CTs were performed that enabled a good visualization of the brain and the cerebellum. Artifacts were limited since most of the big brain/cerebellar fragments had been surgically removed. Therefore, we are confident that our parenchyma evaluation was correct. Moreover, multiple VHIS study results (using the same CTs) were reproduced using MRI by other teams that support our methodology. Finally, the a posteriori evaluation of the involvement of the DN on a template and not on the participant's own neuroimaging might have underestimated its incidence. However, the MNI T2 high definition template used, is a standard template that is typically used in many neuroimaging studies.

Prior studies using VHIS subjects demonstrated that if an area is important for the function or the behavior studied, even 45 years post-injury, deficits are detected and related to burden by caregivers (10, 11). Moreover, studying participants with pTBI evaluated long after the trauma ensured that we evaluated the primary effect of a lesion to a particular area of the brain. This contrasts with the case of progressive central nervous system diseases affecting the cerebellum and other structures (cerebellar ataxia, Spinocerebellar atrophy) or tumors (that might have benefitted from oncological treatment with long lasting side effects beyond the borders of the cerebellum). It can also be difficult to compare cerebellar patients with Stroke to pTBI. The ischemic stroke model is limited by the fact that even if a focal ischemic lesion is seen on neuroimaging, the patient usually suffers from a global neurovascular disease (the exception being an embolic stroke). The effect of this global neurovascular disease is difficult to assess. In pTBI, especially for the participants in this VHIS study, who suffered from their trauma when they were young (18–25 years old), no general neurovascular disease was present at the time of, nor induced by, the injury.

## Conclusions

Our evaluation of male adults with a prior cerebellar pTBI showed only modest working memory and face recognition impairments that were detected only when the cerebellar injury also involved large supra-tentorial brain damage. Other aspects of EF and emotion processing were spared. Notably, the caregivers of cerebellar patients reported no additional burden nor complaints compared to HC caregivers.

In regard to our results, focal cerebellar cortical injury, mostly located in the posterior cerebellum and without damage to the deep cerebellar nuclei does not lead to an impairment in EFs and emotion processing. This suggested that the cerebellar cortex may not be critical for these functions. Rather, the cerebellar cortex may simply be a supportive hub in the neural network that executes these essential high-order behaviors in humans. Studies of focal cerebellar injuries, that cover more broadly the cerebellar cortex, including injury to the deep cerebellar nuclei are needed to complete the interpretation of the role of the cerebellum as crucial or supportive in EFs and emotional processing.

## Data Availability Statement

The raw data supporting the conclusions of this article will be made available by the authors upon reasonable request, without undue reservation.

## Ethics Statement

The studies involving human participants were reviewed and approved by National Institutes of Health Neuroscience Institutional Review Board, Bethesda Naval Hospital and Department of Defense Institutional Review Boards The Institutional Review Board at Northwestern University. The patients/participants provided their written informed consent to participate in this study.

## Author's Note

Questions concerning the Vietnam Head Injury Study can be directed to Dr. Jordan Grafman, jgrafman@northwestern.edu.

## Author Contributions

P-AB designed and performed research, analyzed and interpreted the data, wrote the paper, and performed the statistical analysis. SC-Z, GS, FK, and BG designed and performed research, revised the manuscript. JG designed and performed research, analyzed and interpreted the data, acquired the data, wrote the paper, supervised the study, and performed the statistical analysis. All authors contributed to the article and approved the submitted version.

## Conflict of Interest

The authors declare that the research was conducted in the absence of any commercial or financial relationships that could be construed as a potential conflict of interest.
