# Supplementary material for: A New Insight on the Role of the Cerebellum for Executive Functions and Emotion Processing in Adults
Source: Front Neurol. 2020 Dec 23;11:593490. doi: 10.3389/fneur.2020.593490 (PMC7786249; doi:10.3389/fneur.2020.593490)
Supplement: Supplementary file 1 [file Data_Sheet_1.docx]

**Methods: Neuropsychological testing**

**Delis-Kaplan Executive Function System (D-KEFS) 1:**

**- D-KEFS Trail Making Test—**The D-KEFS Trail Making Test measures the examinee’s cognitive task switching ability, which is an important ability needed for multitasking, simultaneous processing, and divided attention and represents one aspect of cognitive flexibility. The test consists of a visual cancellation task and series of connect-the-circle- tasks. The primary executive function task is the number–letter switching condition, which is a means of assessing flexibility of thinking on a visual-motor sequencing task. As the dependent variable, we used the number–letter switching completion time score, which is the number of seconds that the examinee takes to complete the number–letter switching condition.

**- D-KEFS Verbal Fluency Test—**The D-KEFS Verbal Fluency Test measures the examinee’s word fluency in an effortful, phonemic format. The examinee is asked to generate words that begin with a particular letter as quickly as possible with each 60 s trial. As the dependent variable, we used the letter fluency total correct score, which represents the number of correct words generated for each trial.

**- D-KEFS Sorting Test—**The D-KEFS Sorting Test measures the examinee’s problem-solving behavior (i.e., the verbal/nonverbal modality-specific problem-solving skills, ability to transfer sorting concepts into action, and ability to inhibit previous description responses to engage in flexibility of thinking). This subtest includes two conditions. In the free sorting condition, the examinee is presented with mixed-up cards that display both stimulus words and various perceptual features. The examinee is asked to sort the cards into groups, according to as many different categorization rules, or concepts as possible and to describe the concepts used to generate each sort. In the sort recognition condition, the same sets of cards are each sorted by the examiner into two groups. After each sort made by the examiner, the examinee attempts to identify the correct categorization rule or concept used to generate the sort. As the dependent variable, we used the combined free sorting and sort recognition description score, which is based on the sum of correct description scores in the free sorting and sort recognition conditions.

**- D-KEFS Twenty Question Test—**The D-KEFS Twenty Question Test measures the examinee’s ability of reasoning (i.e., to perceive categories, formulate abstract yes/no questions, and incorporate feedback information into proceeding questions). The examinee is given illustrations of common objects and must ask the fewest number of yes/no questions possible to identify an unknown target object. The fewer yes/no questions an examinee asks, the better his performance on the test. As the dependent variable, we used the total questions asked score, which is based on the number of yes/no questions asked until the target object has been identified.

**- D-KEFS Tower Test—**The D-KEFS Tower Test measures the examinee’s ability for spatial planning. The objective is to move disks that vary in size from small to large across three vertical pegs to construct a designated tower displayed pictorially, in the fewest number of moves possible. Participants must also follow two rules while constructing the tower: they are allowed to move only one disk at a time and they must never place a larger disk over a smaller disk. As the dependent variable, we used the total achievement score, which measures the correct number of constructed towers.

**Wechsler Adult Intelligence Scale-IV (WAIS-IV) 2:**

**- WAIS-IV Working Memory Index—**The Working Memory Index measures the ability to register, maintain, and manipulate auditory information in consciousness awareness. Registration requires attention, auditory and visual discrimination, and concentration. Maintenance is the process by which information is kept active in conscious awareness, using the phonological loop or visual sketchpad. Manipulation is mental re-sequencing of information based on the application of a specific rule.

The Working Memory Index includes the following subtests: Digit Span, Arithmetic, and/or Letter-Number Sequencing. The Digit Span is a verbal/auditory task that measures short-term auditory memory and focus. The task requires the individual to immediately repeat series of numbers of increasingly length that are spoken by the examiner. The individual is required to repeat numbers in order (Forward), in reverse order (Backward), and from lowest to highest (Sequencing). The task measures how long an individual can pay attention to what they hear and “hold” the information in short-term memory long enough to immediately recite the information back or to perform a simple operation with the information (such as re-ordering the numbers). There is no visual information presented or motor response required. The Arithmetic is a verbal task that measures immediate memory and focus during a task that requires the ability to perform mathematical calculations. The examiner reads word problems and the individual is required to complete the calculation “in their head” without the use of paper and pencil. Arithmetic measures attention and memory, but also quick recall of math facts and functions and general proficiency with basic math calculations. There is no visual information or motor response required. The Number-Letter Sequencing **i**s a supplementary verbal task that requires individuals to reorder a series of letters and numbers based a set of specific rules (Letter-Number Sequencing). This task measures short-term memory, attention, and the ability to manipulate/reorder information in short term memory. This task places moderate demands on receptive language skills since the individual must understand the directions and “rules” of the task in order to complete it. There is no visual information or motor response required.

**Facial Expression of Emotion Test (FEEST)** 3:

- The FEEST is a computerized test that assesses recognition of basic emotions (anger, disgust, fear, happiness, sadness, and surprise with stimuli of graded difficulty) via series of pictures of faces

**Mayer-Salovey-Caruso Emotional Intelligence Test (MSCEIT) 4**:

**-** The MSCEIT is the gold-standard emotional intelligence ability-based measure. It assesses emotional intelligence, measuring a person’s capacity for reasoning with emotional information. It is an ability-based scale which measures how well people perform tasks and solve emotional problems, rather than simply asking them, for example, about their subjective assessment of their emotional skills. It consists of 141 items that are assessing 4 domains of emotion : Perceiving Emotions (the ability to perceive emotions in oneself and others as well as in objects, art, stories, music, and other stimuli) ; Facilitating Thought (the ability to generate, use, and feel emotion as necessary in the communication of feelings or employing them in other cognitive processes) ;Understanding Emotions (the ability to understand emotional information, to understand how emotions combine and progress through relationship transitions, and to appreciate emotional meanings) ;Managing Emotions (the ability to be open to feelings, and to modulate them in oneself and others so as to promote personal understanding and growth). Perceiving Emotion and Using Emotion scores are grouped to make the Experimental Emotion Intelligence Score. Understanding Emotion and Managing Emotion are grouped to make the Strategic Emotion Intelligence Score. Both of these scores are grouped to make the Overall Emotion Intelligence Score.

**The Vocal Emotional Task 5,6:**

- The Vocal Emotional Task Assesses recognition of vocal emotional sounds. As the dependent variable, we used the error rate, which is based on the total number of errors for all the different vocal emotions.

**The Zarit Burden Interview 7**:

- The Burden Interview has been specially designed to reflect the stresses experienced by caregivers of patients. It can be completed by caregivers themselves or as part of an interview. Caregivers are asked to respond to a series of 22 questions about the impact of the patient’s disabilities on their life. For each item, caregivers are to indicate how often they felt that way (never, rarely, sometimes, quite frequently, or nearly always). The Burden Interview is scored by adding the numbered responses of the individual items. Higher scores indicate greater caregiver distress.


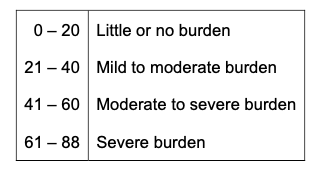


**The Frontal System Behavior Scale (FrSBe) 8**:

- The FrSBe is designed to measure changes in behavior as a consequence of frontal systems dysfunction. The FrSBe is a 46-item rating scale, with three subscales: Apathy (14 items), Disinhibition (15 items) and Executive dysfunction (17 items). Item content of the Apathy scale samples “problems with initiation, psychomotor retardation, spontaneity, drive, persistence, loss of energy and interest, lack of concern about self/care, and/or blunted affective expression”. The Disinhibition subscale items assess problems with inhibitory control of actions and emotions, including impulsivity, hyperactivity, social inappropriateness, emotional liability, explosiveness, irritability. Problem areas addressed in the Executive dysfunction subscale include “sustained attention, working memory, organization, planning, future orientation, sequencing, problem solving, insight, mental flexibility, self-monitoring of ongoing behavior, and/or the ability to benefit from feedback or modify behavior following errors. The Self-rating and Family forms have identical items, phrased as appropriate. Items are rated in a 5-point scale: 1 (almost never), 2 (seldom), 3 (sometimes), 4 (frequently), 5 (almost always). Four scores are obtained: Total, Apathy, Disinhibition and Executive. Scores greater than T = 65 are considered clinically significant.

**Methods: Computed Tomography image acquisition and analysis**

During Phase 3, we acquired axial computed tomography (CT) scans without contrast of participants at the Bethesda Naval Hospital on a General Electric Medical Systems Light Speed Plus CT scanner in helical mode. Images were reconstructed with an in-plane voxel size of 0.4 x 0.4 mm, an overlapping slice thickness of 2.5 mm and 1-mm slice interval. We determined lesion location and volume from CT images using the Analysis of Brain Lesion (ABLe v.2.8b) software (Makale *et al.*, 2002; Solomon *et al.*, 2007) that is implemented within MEDx v.3.44 (Medical Numerics, Germantown, MD, USA). Each cortical and cerebellar anatomical structures were differentiate using the Automated anatomical Labelling (AAL) atlas 9. For the cerebellum, 8 region of interests (ROIs) were used for the Vermis (I-II, III, IV-V, VI, VII, VIII, IX, X) and 9 ROIs were used in each cerebellar hemisphere (lobule III, IV-V, VI, Crus I, Crus II, VIIb, VIII, IX, X). We spatially normalized each CT image to a CT template brain image in Montreal Neurological Institute (MNI) space 10. We performed a spatial normalization with the automated image registration (AIR) algorithm 11, using a 12-parameter affine fit. Lesion volume was calculated by manually tracing the lesion in all relevant slices of the CT image, and then summing the traced areas and multiplying by slice thickness. A trained neuropsychiatrist performed the manual tracing, which was then reviewed by an observer (JG) blind to the results of the clinical evaluation and neuropsychological assessment. A board-certified neurologist (Andres M. Salazar, M.D.) experienced in reading brain scans and evaluating pTBI participants blindly reviewed all the images and confirmed that no additional gray and white matter damage, stroke or additional injuries occurred post-injury.

**Methods: Statistical Analyses**

JASP is a publicly available software developed and supported by the University of Amsterdam. The inferential engine is based on R 12 and –for the Bayesian analyses– much use is made of the “BayesFactor “package developed by Morey and Rouder 13 and the “conting” package developed by Overstall and King 14. It is a robust and valid software that has already been used in multiple publications 15–19. To analyze the Bayesian factor (BF), we used the JASP classification 20, which is an adaption of Jeffery’s scheme 21. The Bayes factor (BF) represents an odds ratio, i.e., the probability of the data under one hypothesis relative to another. BF values can be considered “anecdotal”, “moderate”, “strong”, “very strong”, or “extreme” relative evidence for a hypothesis depending on the value 18. BF10 assesses the level of evidence in favor of the alternative hypothesis (H1) relative to the null hypothesis (H0) 18. As example, a value of BF10 = 8 designates the data are eight times more likely under H1 than H0. When BF10 is between 1 and 3, negligible/anecdotal evidence for the alternative hypothesis can be assumed, between 3 and 10, moderate evidence for the alternative hypothesis can be assumed, and above 10 strong evidence for the alternative hypothesis can be assumed 20. As in previous studies, the most common prior model was used, as the default in JASP software 22,23. This model places data points in realistic ranges without being overcommitted to any one point. In addition, it has a moderate size effect, fits a large set of psychological data, and carry a minimum degree of information 24,25.

**REFERENCES**

1 Delis D, Kaplan E, Kramer J. Delis-Kaplan Executive Function System, The Psychological Corporation. San Antonio, 2001.

2 Wechsler D. Manual for the Wechsler Adult Intelligence Scale-Revised. 1981.

3 Young A, Perrett DI, Calder A, Sprengelmeyer RH, Ekman P. Facial expressions of emotion: Stimuli and Test (FEEST). 2002. http://www.harcourt-uk.com/product.aspx?skey=2835.

4 Mayer JD, Caruso DR, Salovey P. Emotional intelligence meets traditional standards for an intelligence. *Intelligence* 1999; **27**: 267–98.

5 Hornak J, Rolls ET, Wade D. Face and voice expression identification in patients with emotional and behavioural changes following ventral frontal lobe damage. *Neuropsychologia* 1996; **34**: 247–61.

6 Hornak J, Bramham J, Rolls ET, *et al.* Changes in emotion after circumscribed surgical lesions of the orbitofrontal and cingulate cortices. *Brain J Neurol* 2003; **126**: 1691–712.

7 Zarit SH, Reever KE, Bach-Peterson J. Relatives of the impaired elderly: correlates of feelings of burden. *The Gerontologist* 1980; **20**: 649–55.

8 Stout JC, Ready RE, Grace J, Malloy PF, Paulsen JS. Factor analysis of the frontal systems behavior scale (FrSBe). *Assessment* 2003; **10**: 79–85.

9 Tzourio-Mazoyer N, Landeau B, Papathanassiou D, *et al.* Automated anatomical labeling of activations in SPM using a macroscopic anatomical parcellation of the MNI MRI single-subject brain. *NeuroImage* 2002; **15**: 273–89.

10 Collins DL, Neelin P, Peters TM, Evans AC. Automatic 3D intersubject registration of MR volumetric data in standardized Talairach space. *J Comput Assist Tomogr* 1994; **18**: 192–205.

11 Woods RP, Grafton ST, Holmes CJ, Cherry SR, Mazziotta JC. Automated image registration: I. General methods and intrasubject, intramodality validation. *J Comput Assist Tomogr* 1998; **22**: 139–52.

12 Team RC. R: A language and environment for statistical computing. 2013.

13 Morey RD, Rouder JN. BayesFactor 0.9. 12-2. Comprehensive R Archive Network. 2015.

14 Overstall A, King R. conting: An R package for Bayesian analysis of complete and incomplete contingency tables. *J Stat Softw* 2014; **58**: 1–27.

15 Nuzzo RL. An Introduction to Bayesian Data Analysis for Correlations. *PM R* 2017; **9**: 1278–82.

16 Gu X, Mulder J, Hoijtink H. Approximated adjusted fractional Bayes factors: A general method for testing informative hypotheses. *Br J Math Stat Psychol* 2018; **71**: 229–61.

17 Perezgonzalez JD, Frías-Navarro MD. Retract p < 0.005 and propose using JASP, instead. *F1000Research* 2018; **6**. DOI:10.12688/f1000research.13389.2.

18 Quintana DS, Williams DR. Bayesian alternatives for common null-hypothesis significance tests in psychiatry: a non-technical guide using JASP. *BMC Psychiatry* 2018; **18**: 178–178.

19 Wagenmakers E-J, Love J, Marsman M, *et al.* Bayesian inference for psychology. Part II: Example applications with JASP. *Psychon Bull Rev* 2018; **25**: 58–76.

20 lee M, Wagenmakers E. Bayesian cognitive modeling: a practical course. Cambridge: Cambridge University Press, 2014.

21 Jeffreys H. The theory of probability. Oxford: Oxford University Press, 1961.

22 Hoekstra R, Monden R, van Ravenzwaaij D, Wagenmakers E-J. Bayesian reanalysis of null results reported in medicine: Strong yet variable evidence for the absence of treatment effects. *PloS One* 2018; **13**: e0195474–e0195474.

23 Matsugi A, Yoshida N, Nishishita S, *et al.* Cerebellum-mediated trainability of eye and head movements for dynamic gazing. *PloS One* 2019; **14**: e0224458–e0224458.

24 Rouder JN, Speckman PL, Sun D, Morey RD, Iverson G. Bayesian t tests for accepting and rejecting the null hypothesis. *Psychon Bull Rev* 2009; **16**: 225–37.

25 Rouder JN, Morey RD, Speckman PL, Province JM. Default Bayes factors for ANOVA designs. *J Math Psychol* 2012; **56**: 356–74.

**suppl-Table 1: Motor measures by veterans with cerebellar damage and veterans with no head injury (Healthy control) using the D-KEFS**

|  | **Cerebellar Group** | **Healthy Control** | **Statistics** |
| --- | --- | --- | --- |
| **Paresis (Y,N)**** | 7, 17 | 0, 54 | *Χ*^2^_(1, N = 78)_ = 17.303, p <.001, BF_10_=1046.92 |
| **Ataxia(Y,N)** | 2, 22 | 1, 54 | *Χ*^2^_(1, N = 79)_ = 1.941, p=0.164, BF_10_=1.35 |
| **Gait Abnormalities**  **(Y,N)**** | 6, 18 | 1, 54 | *Χ*^2^_(1, N = 79)_ = 11.119, p <.001, BF_10_=53.64 |
| **Voluntary Movement Abnormalities**  **(Y,N)**** | 8, 16 | 1, 54 | *Χ*^2^_(1, N = 79)_ = 16.440, p <.001, BF_10_=573.29 |
| **Station Posture Impairment(Y,N)** | 2, 22 | 1, 53 | *Χ*^2^_(1, N = 78)_ = 1.887, p=0.169, BF_10_=1.33 |
| **Muscle Tone Impairement(Y,N)**** | 3, 21 | 0, 55 | *Χ*^2^_(1, N = 79)_ = 7.146, p=0.008, BF_10_=10.15 |
| **Purdue Pegboard:Both Hands**** | M=10.25 (SD=2.95) | M=11.94 (SD=1.16) | U=199.5, p=0.01, RBC=-.377, BF_10_=3.62 |
| **Purdue Pegboard:Assembly**  ****** | M=32.60 (SD=8.97) | M=37.44 (SD=4.5) | t_(50)_=-2.501, p=0.008, d=-.713, BF_10_=2.84 |

Y: Yes, N: No, RBC: Rank-Biserial Correlation, M: Mean, SD: Standard Deviation, AFQT: Armed Forces Qualification Test

** significantly different p<0.05

Perdue Pegboard scores are the number of pin placed.

**suppl -Table 2: Neuropsychological measures of executive functions completed by veterans with cerebellar damage and veterans with no head injury (Healthy control) using the D-KEFS**

|  | **Cerebellar Group** | **Healthy Control** | **Statistics** |
| --- | --- | --- | --- |
| **D-KEFS Trail Making Test** | M=8.61 (SD=3.68) | M=9.63 (SD=3.25) | U=516.5, p=0.121, RBC=-.168, BF10=0.67 |
| **D-KEFS Verbal Fluency Test** | M=9 (SD=4.06) | M=11.11 (SD=3.67) | t(75)=-1.179, p=.121 d=-.29, BF10=0.79 |
| **D-KEFS Sorting Test** | M=20.87 (SD=5.07) | M=21.62 (SD=5.24) | t(75)=-0.588, p=.279, d=-.146, BF10=0.41 |
| **D-KEFS Twenty Question Test** | M=9.83 (SD=3.1) | M=10.18 (SD=3.27) | U=538, p=0.177, RBC=-.134, BF10=0.34 |
| **D-KEFS Tower Test** | M=10.61 (SD=2.25) | M=11.26 (SD=2.66) | t(77)=-1.027, p=.154, d=-.026, BF10=0.66 |

M: Mean, SD: Standard Deviation

D-KEFS: Delis Kaplan Executive Function System

**suppl -Table 3: Neuropsychological measures of emotional processes completed by veterans with cerebellar damage and veterans with no head injury (Healthy control) using the Mayer-Salovey-Caruso-Emotional-Intelligence-Test**

|  | **Cerebellar Group** | **Healthy Control** | **Statistics** |
| --- | --- | --- | --- |
| **Perceiving Emotion** | M=101.19 (SD=29.99) | M=109.102 (SD=29.24) | U=453.5, p=0.203 RBC=-.128, BF10=0.60 |
| **Using Emotion** | M=94.69 (SD=24.68) | M=105.06 (SD=20.15) | t(70)=-1.835, p=.035 d=-.48, BF10=2.04 |
| **Understanding Emotion** | M=87.31 (SD=14.29) | M=95.77 (SD=16.79) | U=353.5, p=0.018 RBC=-.320, BF10=2.19 |
| **Managing Emotion** | M=93.54 (SD=11.26) | M=93.04 (SD=14.29) | U=536.5, p=0.585 RBC=.03, BF10=0.20 |
| **Experimental Emotion Intelligence Score** | M=93.09 (SD=19.21) | M=100.83 (SD=19.01) | t(70)=-1.543, p=.064 d=-.41, BF10=1.32 |
| **Strategic Emotion Intelligence Score** | M=89.02 (SD=13.33) | M=94.81 (SD=17.15) | U=418.5, p=0.102, RBC=-.195, BF10=0.93 |
| **Overall Emotion**  **Intelligence Score** | M=88.50 (SD=17.93) | M=95.04 (SD=16.07) | U=410.5, p=0.085, RBC=-.211, BF10=1.053 |

M: Mean, SD: Standard Deviation

**suppl -Table 4: Cognitive and Behavior Burden and Complaints measures completed by relatives of veterans with cerebellar damage and veterans with no head injury (Healthy control)**

|  | **Cerebellar Group** | **Healthy Control** | **Statistics** |
| --- | --- | --- | --- |
| **Zarit Burden Interview** | M=14.12 (SD=15.21) | M=8.90 (SD=6.12) | U=256, p=0.649 RBC=.067, BF10=0.25 |
| **FrSBE Apathy** | M=64 (SD=25.08) | M=58.56 (SD=18.19) | U=232.5, p=0.666 RBC=.076, BF10=0.23 |
| **FrSBE Disinhibition** | M=87.31 (SD=14.29) | M=95.77 (SD=16.79) | U=184, p=0.214 RBC=-.148, BF10=0.41 |
| **FrSBE Executive Dysfunction** | M=93.54 (SD=11.26) | M=93.04 (SD=14.29) | U=224.5, p=0.590 RBC=.039, BF10=0.23 |
| **FrSBE Total** | M=93.09 (SD=19.21) | M=100.83 (SD=19.01) | U=209.5, p=0.440 RBC=-.03, BF10=0.27 |

FrSBE: Frontal Systems Behavior Scale; M: Mean; SD: Standard Deviation
